# Supplementary material for: Transcriptomic Insights into Mechanisms of Early Seed Maturation in the Garden Pea (Pisum sativum L.)
Source: Cells. 2020 Mar 23;9(3):779. doi: 10.3390/cells9030779 (PMC7140803; doi:10.3390/cells9030779)

Sprint−2,20(3)

Global Summary

%DE = 0.23

genes with fdr < 0.2 = 6284 (2715 + / 3569 −)

genes with fdr < 0.1 = 5877 (2561 + / 3316 −)

genes with fdr < 0.05 = 5466 (2389 + / 3077 −)

genes with fdr < 0.01 = 4954 (2187 + / 2767 −)

genes in genesets = 0

<FC> = 0

<t−score> = 0

<p−value> = 0

<fdr> = 0.77

Portrait

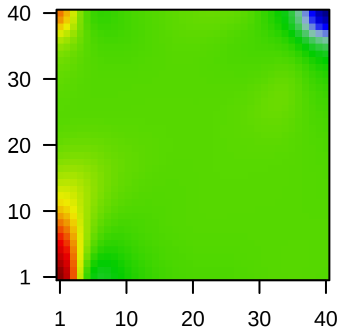

Top 100 DE genes

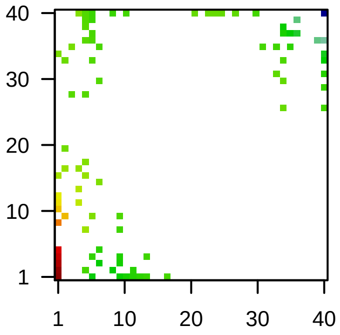

Global Genelist

Rank	ID	log(FC)	p−value / fdr / Metagene	Description
1	TRINITY_DN2	0.05	0.0000000[illegible]00002	
2	TRINITY_DN2	−0.06	0.0000000[illegible]00002	
3	TRINITY_DN1	0.26	0.0000000[illegible]00002	
4	TRINITY_DN4	−0.04	0.0000000[illegible]00002	
5	TRINITY_DN4	−0.11	0.0000000[illegible]00002	
6	TRINITY_DN3	0.04	0.0000000[illegible]00002	
7	TRINITY_DN3	−0.03	0.0000000[illegible]00002	
8	TRINITY_DN3	−0.04	0.0000000[illegible]00002	
9	TRINITY_DN3	0.07	0.0000000[illegible]00002	
10	TRINITY_DN3	−0.04	0.0000000[illegible]00002	
11	TRINITY_DN3	0.05	0.0000000[illegible]00002	
12	TRINITY_DN3	−0.04	0.0000000[illegible]00002	
13	TRINITY_DN3	0.12	0.0000000[illegible]00002	
14	TRINITY_DN1	0.78	0.0000000[illegible]00002	
15	TRINITY_DN1	−0.03	0.0000000[illegible]00002	
16	TRINITY_DN1	−0.12	0.0000000[illegible]00002	
17	TRINITY_DN1	−0.03	0.0000000[illegible]00002	
18	TRINITY_DN1	0.04	0.0000000[illegible]00002	
19	TRINITY_DN1	−0.06	0.0000000[illegible]00002	
20	TRINITY_DN1	−0.04	0.0000000[illegible]00002	

p−values

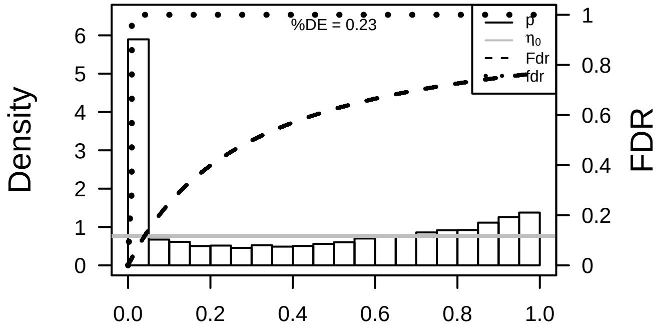

Supplement: Supplementary file 1 [file cells-09-00779-s001.zip › Supplementary materials/FileS2.zip!/FileS1/Summary Sheets - Samples/Sprint.2.20.3..pdf]
